# PrLPAAT4, a Putative Lysophosphatidic Acid Acyltransferase from *Paeonia rockii*, Plays an Important Role in Seed Fatty Acid Biosynthesis

**DOI:** 10.3390/molecules22101694

**Published:** 2017-10-10

**Authors:** Qingyu Zhang, Rui Yu, Daoyang Sun, Zhangzhen Bai, Hong Li, Liang Xue, Yanlong Zhang, Lixin Niu

**Affiliations:** 1 College of Landscape Architecture and Arts, Northwest A&F University, Yangling, Shaanxi 712100, China; zhangqingyu@nwsuaf.edu.cn (Q.Z.); yurui@nwsuaf.edu.cn (R.Y.); sundaoyang2009@163.com (D.S.); baizhangzhen@nwsuaf.edu.cn (Z.B.); 2 Institute of Biochemistry and Cell Biology, Shanghai Institutes for Biological Sciences, Chinese Academy of Sciences, Shanghai 200031, China; hong.li@sibcb.ac.cn (H.L.); xueliang@sibcb.ac.cn (L.X.)

**Keywords:** lysophosphatidic acid acyltransferase, triacylglycerol, tree peony, Arabidopsis, oleic acid, fatty acid

## Abstract

Lysophosphatidic acid acyltransferases (LPAATs) are essential for the acylation of lysophosphatidic acid (LPA) and the synthesis of phosphatidic acid (PA), a key intermediate in the synthesis of membrane phospholipids and storage lipids. Here, a putative lysophosphatidic acid acyltransferase gene, designated *PrLPAAT4*, was isolated from seed unsaturated fatty acid (UFA)-rich *P. rockii*. The complete *PrLPAAT4* cDNA contained a 1116-bp open reading frame (ORF), encoding a 42.9 kDa protein with 371 amino acid residues. Bioinformatic analysis indicates that PrLPAAT4 is a plasma membrane protein belonging to acyl-CoA:1-acylglycerol-sn-3-phosphate acyltranferases (AGPAT) family. PrLPAAT4 shared high sequence similarity with its homologs from *Citrus clementina*, *Populus trichocarpa*, *Manihot esculenta*, and *Ricinus communis*. In Arabidopsis, overexpression of *PrLPAAT4* resulted in a significant increase in the content of oleic acid (OA) and total fatty acids (FAs) in seeds. *AtDGAT1*, *AtGPAT9*, and *AtOleosin*, involved in TAG assembly, were upregulated in *PrLPAAT4*-overexpressing lines. These results indicated that PrLPAAT4 functions may be as a positive regulator in seed FA biosynthesis.

## 1. Introduction

Triacylglycerol (TAG) is a critical form of oil storage in many seeds by providing energy and carbon to support the post-germination seedling establishment [1]. Fatty acids (FAs) are mainly synthesis de novo from acyl-CoAs in plastids, and then transported to the endoplasmic reticulum (ER) or cytoplasm [2]. TAG is synthesized from free FAs and glycerol in the ER through the Kennedy pathway, which sequentially transfer acyl chains from acyl-CoAs to the sn-1, -2, and -3 positions of the glycerol-3-phosphate (G3P) backbone via three enzymes: glycerol-3-phosphate acyltransferase (GPAT), lysophosphatidic acid acyltransferase (LPAAT), and diacylglycerol acyltransferase (DGAT) [3,4,5]. Furthermore, TAG can be synthesized through the transacylation of sn-2 FA from phospatidylcholine (PC) onto sn-3-position of diacylglycerols (DAG), which is catalyzed by the phospholipid: diacylglycerol acyltransferase (PDAT) [6,7].

LPAATs play a crucial role in catalyzing the second step of TAG formation, determining TAGs acyl composition at the sn-2-position, and controlling the conversion of LPA to PA [8,9]. Since the initial report of LPAAT activities, a number of LPAAT genes have been cloned and characterized in *Zea mays* [10], *Cocos nucidera* [11], *Brassica napus* [12], *Arabidopsis thaliana* [13], *Tropaeolum majus* [14], *Arachis hypogaea* [15], and *Ricinus communis* [16]. Previous studies have demonstrated that LPAATs contribute to the regulation of many physiological processes, especially seed oil biosynthesis.

In Arabidopsis, overexpression of two microsomal LPAAT genes, *BAT1.13* and *BAT1.5* from *B. napus*, increases oil content and seed mass [17]. When the yeast gene *SLC1* with LPAAT activity was constitutively expressed in soybean, the transgenic plants displayed elevated seed oil content [18]. Overexpression of *SLC1-1*, another putative LPAAT gene from the yeast, in Arabidopsis and *B. napus* leads to a substantial increase in the proportion of very-long-chain FA at the sn-2 position of seed TAG and content of seed oil from 8% to 48% [19]. Moreover, LPAATs are also associated with various membrane systems, including chloroplasts, ER, and the outer membrane of mitochondria [4,20]. In the Arabidopsis genome, a total of five LPAAT genes have been identified and designated, including one plastidial isoenzyme gene *AtLPAAT1*, and four cytoplasmatic isoenzyme genes *AtLPAAT2*, *AtLPAAT3*, *AtLPAAT4*, and *AtLPAAT5* [20,21]. *AtLPAAT1*, *AtLPAAT2*, and *AtLPAAT3* are shown to be essential for normal plant development, and *AtLPAAT4* and *AtLPAAT5* cause alternative transcripts mostly differing in the 5′-UTR, suggesting a high complexity in PA synthesis [22]. To date, however, the roles of each LPAAT isoform in the biosynthesis of seed FA are still not completely known.

Tree peony (*Paeonia* section *Moutan* DC.), possessing striking ornamental and medicinal value, is a deciduous shrub native to China [23,24,25]. Its seeds contain relatively high amounts of oil, approximately 90% of which is unsaturated fatty acids (UFAs), such as oleic acid (OA), linoleic acid (LA), and A-linolenic acid (ALA) [26,27]. Accordingly, the tree peony seed has been revealed to have a great potential for production of edible oil [28]. As a novel oil crop, the molecular regulatory mechanism of seed oil biosynthesis in tree peony remains unknown. In our earlier studies, we have identified a *P. rockii* LPAAT gene, *PrLPAAT1*, whose ectopic overexpression in Arabidopsis remarkably increased the accumulation of total FAs and main FA ingredients. Additionally, we have found that the tissue-specific expression profile of *PrLPAAT4* was similar with that of *PrLPAAT1*, and they have relatively high transcript abundances in floral tissues and developing seeds at the early developmental stages [29]. In this study, we focused on the impact of *PrLPAAT4* on seed FA biosynthesis. We found that transgenic Arabidopsis plants seed-specifically expressing *PrLPAAT4* showed increased content of total FAs and OA, as well as upregulated transcripts of TAG biosynthesis-related genes. Our work could be useful to better understand the molecular mechanism of lipid biosynthesis in the high levels of UFA-containing tree peony seed.

## 2. Results

### 2.1. Isolation and Characterization of PrLPAAT4

**Figure 1:**
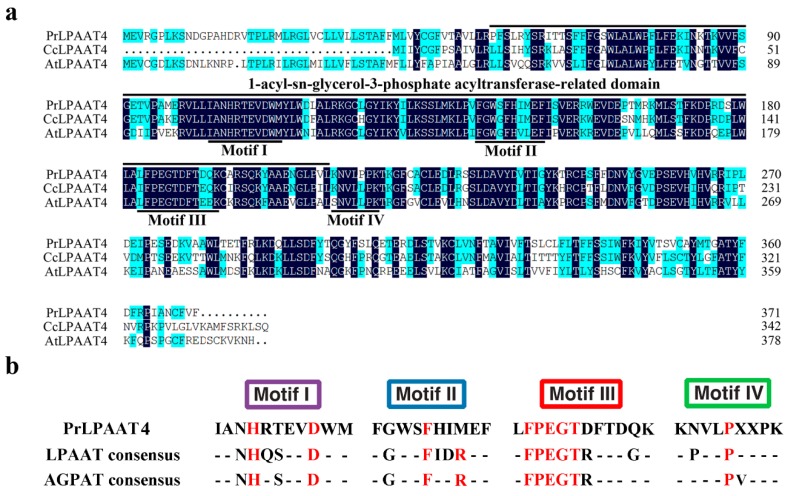
Sequence analysis of PrLPAAT4. (**a**) alignment of deduced PrLPAAT4 amino acid sequence with similar proteins from *Citrus clementina* CcLPAAT4 (XP_006423197) and Arabidopsis AtLPAAT4 (NP_565098). Identical amino acids are *boxed* in *dark bule* and similar amino acids are *shaded* in *light blue*. *Solid lines* indicate the conserved 1-acyl-sn-glycerol-3-phosphate acyltransferase-related domain. The conserved acyltransferase motifs (I–IV) are underlined; (**b**) acyltransferase motifs in PrLPAAT4, LPAATs, and AGPATs. The conserved amino acid residues are shown in red.

Based on the nucleotide sequences of *LPAAT4* homologies from different plant species, specific primers were designed to amplify the partial conserved cDNA sequence of *LPAAT4* gene in *P. rockii*. One fragment with an expected size of 349 bp was obtained from the seeds. Its deduced amino acid (aa) sequences had a high identity to the coding region of other known plant LPAAT4 sequences. Next, full-length cDNA sequence of the LPAAT4 gene from *P. rockii* was amplified through RACE and annotated as *PrLPAAT4* (GenBank accession number: KX256279). The complete *PrLPAAT4* cDNA contained a predicted 1116-bp ORF, which encoded a protein of 371 aa residues (Figure 1a). Analysis of the conserved domains revealed that PrLPAAT4 possesses 1-acyl-sn-glycerol-3-phosphate acyltransferase-related domain, sharing high similarity with CcLPAAT4 in clementine and AtLPAAAT4 in Arabidopsis as shown by amino acid alignment (Figure 1a) Sequence alignment revealed that PrLPAAT4 had four similar conserved acyltransferase motifs (I–IV) with acyl-CoA:1-acylglycerol-sn-3-phosphate acyltranferases (AGPATs) and LPAATs (Figure 1b).

### 2.2. The Predicted Structure of PrLPAAT4

**Figure 2:**
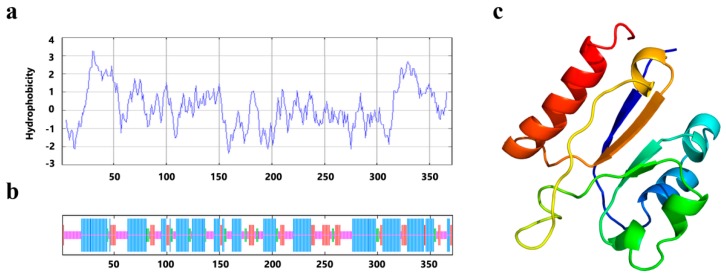
Predicted structure of PrLPAAT4. (**a**) hydrophobicity plot predicted by the Kyte–Doolittle method of a sliding window average over nine neighboring residues; (**b**) the secondary structure of PrLPAAT4. Blue, red, green, and pink lines show the predicted α-helices, extended strands, β-turns, and random coils, respectively; (**c**) tertiary structure of the partial PrLPAAT4 protein. The helical structures show the predicted α-helices, and the arrows indicate the β-sheets.

According to the analysis by ProtParam, the chemical formula for the PrLPAAT4 protein was C1986H3031N489O529S21, and its molecular weight was 42.9 kDa. The instability index (II) of PrLPAAT4 was 44.70, indicating that it belonged to one of the unstable proteins. Hydrophobicity analysis suggested that the PrLPAAT4 protein was a hydrophobic protein with the grand average of hydrophobicity (GRAVY) was 0.170 (Figure 2a). Secondary structure prediction showed that PrLPAAT4 contained 50.67% α-helix, 14.82% β-sheet, 6.47% β-turn, and 28.03% random coil (Figure 2b). Protein transmembrane topology analysis revealed three transmembrane regions in amino acid residues (No. 30~52, 62~79 and 319~341) of PrLPAAT4, which was localized to the outer cell membrane. The finding suggests that PrLPAAT4 was a transmembrane protein. Space prediction of three-dimensional structure showed that the protein consisted of six α-helices and four β-sheets (Figure 2c).

### 2.3. Phylogenetic Analysis of PrLPAAT4

**Figure 3:**
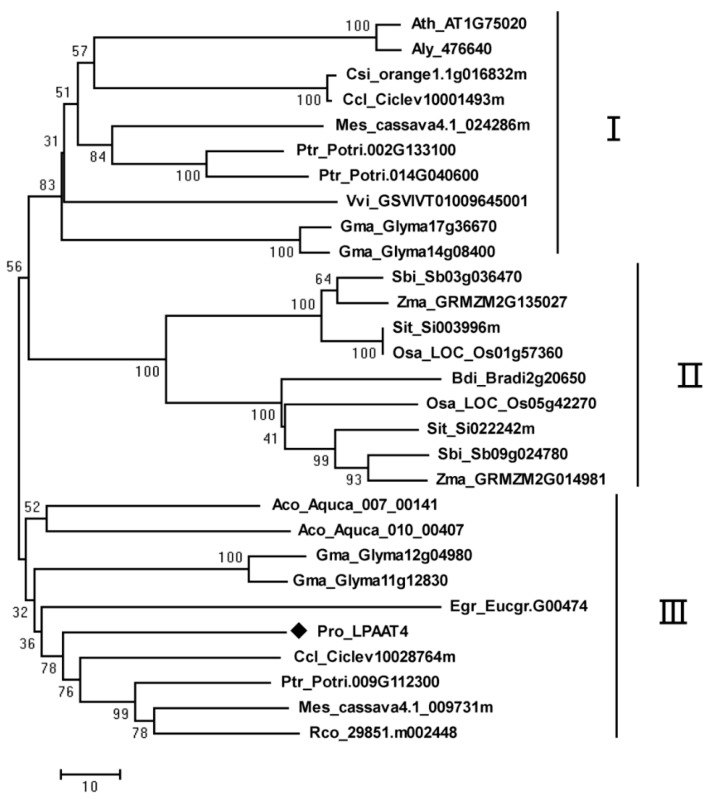
Phylogenetic comparison of the LPAAT4 proteins. Plant species included in the phylogenetic tree are *Manihot esculenta* (Mes), *Ricinus communis* (Rco), *Populus trichocarpa* (Ptr), *Glycine max* (Gma), *Arabidopsis thaliana* (Ath), *Arabidopsis lyrata* (Aly), *Citrus sinensis* (Csi), *Citrus clementina* (Ccl), *Eucalyptus grandis* (Egr), *Vitis vinifera* (Vvi), *Aquilegia coerulea Goldsmithn* (Aco), *Sorghum bicolor* (Sbi), *Zea mays* (Zma), *Setaria italica* (Sit), *Oryza sativa* (Osa), and *Brachypodium distachyon* (Bdi)*.* PrLPAAT4 is highlighted with a rhombus. The neighbor-joining method with MEGA software (version 5.1, Tokyo Metropolitan University, Hachioji, Tokyo, Japan) was used to construct the phylogenic tree. Boot-strap values as a percentage of 1000 replicates are indicated at corresponding branch nodes. *Scale bar* represents the number of amino acid substitutions per site.

To elucidate the phylogenetic relationship of PrLPAAT4 with the other homologues proteins, a phylogenetic tree was constructed with their aa sequences (Figure 3). The LPAAT4 proteins of various species had different length ranging from 343 aa to 406 aa, and two introns were present in their genomic DNA sequences (Table S2). All plant LPAAT4 homologs were divided into three clusters: I and III, dicotyledonous plants; II, monocotyledonous plants (Figure 3). PrLPAAT4 was subordinate to cluster III, and its sequence identities with the similar proteins from *Citrus clementina*, *Populus trichocarpa*, *Manihot esculenta*, and *Ricinus communis* were 73.80%, 73.91%, 71.08%, and 71.10%, respectively.

### 2.4. Comparative Analysis of PrLPAAT4 and Its Deduced Protein Structure

**Figure 4:**
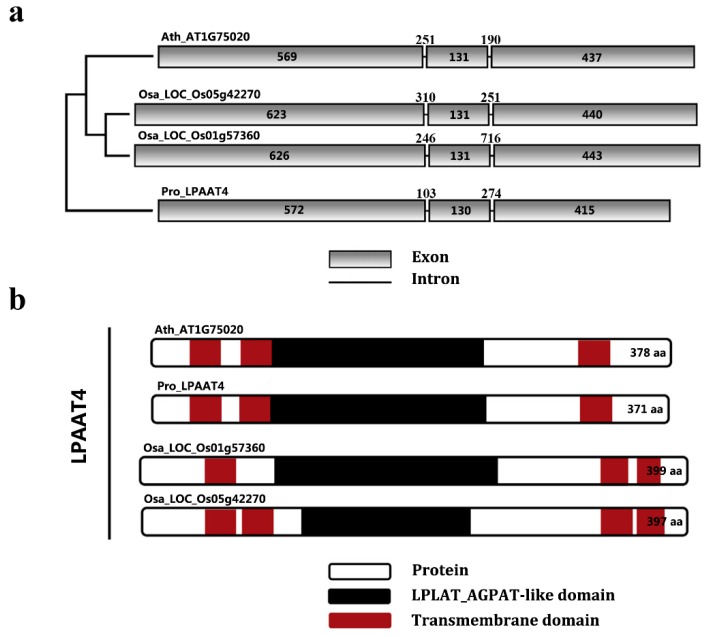
Comparative analysis of gene structure and conserved domain in the LPAAT4 proteins. (**a**) exon–intron structure of plant LPAAT4 genomic DNAs. Representative sequences are presented for each cluster: *A. thaliana* (Ath), *O. sativa* (Osa) and *P.* *rockii* (Pro). The size of each exon and intron is shown by bp; (**b**) prediction of the transmembrane and acyltransferase domains of plant LPAAT4 proteins. Predicted transmembrane structures (*red*) and LPLAT_AGPAT-like domain (*black*) of representative species were obtained using the transmembrane sever TMHMM-2.0 and the SMART database with the complete protein sequences. The sequences are represented as *simplified boxes*. The size of each sequence is shown by aa residues.

Comparative analysis of exons/introns is important for better understanding the gene structure and organization, protein functionality and evolutionary changes among species [30]. Three representative plants, *A. thaliana*, *Oryza sativa*, and *P. rockii*, were selected from cluster I, II, and III, respectively, to map the LPAAT4 genetic structure. All four *LPAAT4* genes were composed of three exons and two introns, sharing high similarity in exon and intron sizes (Figure 4a). Prediction of transmembrane structures revealed that each LPAAT4 protein contained at least three transmembrane domains and one LPLAT_AGPAT-like domain (Figure 4b). In the case of transmembrane domain distribution, PrLPAAT4 was similar with Arabidopsis LPAAT4 protein but different with two rice LPAAT4 proteins (Figure 4b). LPAAT4 proteins from *A. thaliana* and *P. rockii* exhibited an overlap between the transmembrane and LPLAT_AGPAT-like domains (Figure 4b).

### 2.5. PrLPAAT4 Is Localized to the Plasma Membrane

**Figure 5:**
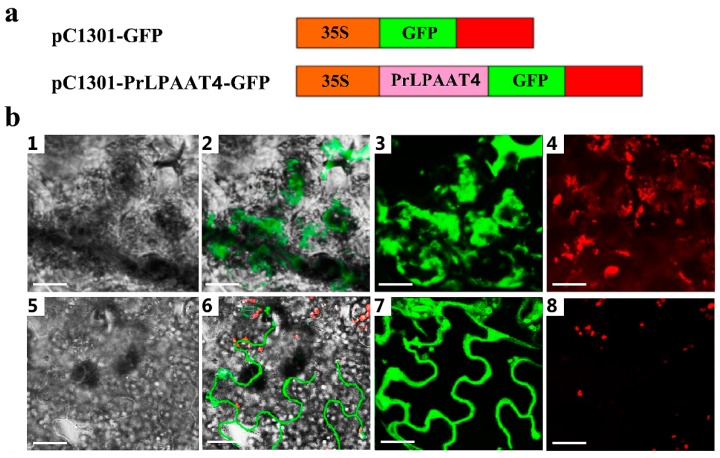
Subcellular localization of PrLPAAT4 in tobacco cells. (**a**) schematic diagram of the construction of the recombinant 35S::*PrLPAAT4*-*GFP* vector. 35S: a constitutive promoter from the cauliflower mosaic virus; GFP: green fluorescent protein; NOS: Nopaline synthase terminator; (**b**) 1–4: GFP florescence detection in tobacco leaves transformed with pC1301-*GFP*; 5–8: GFP florescence detection in tobacco leaves transformed with pC1301-*PrLPAAT4*-*GFP*; 1, 5: bright field images; 3, 7: GFP florescence; 4, 8: Chlorophyll autoflorescence; 2, 6: merging images of others. *Scale bars* = 23.00 μm.

To investigate the subcellular localization of the PrLPAAT4 protein, the plasmid 35S::*PrLPAAT4*-*GFP* (pC1301-PrLPAAT4-GFP) was transiently transformed into the lower epidermis of tobacco leaves by *Agrobacterium*-mediated transformation method, using an empty vector 35S::*GFP* (pC1301-GFP) as a control (Figure 5a). The results showed that the control GFP fluorescence was spread throughout the entire cellular structures, including the nucleus, cytomembrane and cytoplasm (Figure 5b). Nevertheless, the GFP fluorescence by the 35S::*PrLPAAT4*-*GFP* chimera was observed in the plasma membrane of tobacco lower epidermal cells, suggesting the localization of PrLPAAT4 to the plasma membrane (Figure 5b).

### 2.6. Overexpression of PrLPAAT4 in Arabidopsis Increases FA Content and Expression of FA and Lipid Biosynthetic Genes

**Figure 6:**
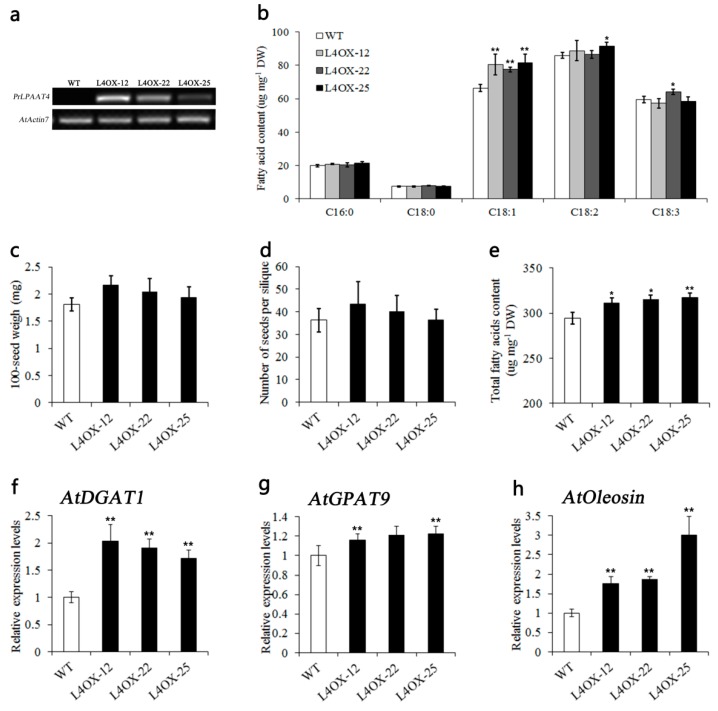
Effect of *PrLPAAT4* overexpression on plant growth, FA content and composition of transgenic Arabidopsis seeds. (**a**) semi-quantitative RT-PCR analysis of *PrLPAAT4* transcript abundances in the seeds of transgenic Arabidopsis lines overexpressing *PrLPAAT4*, compared to WT; (**b**) main FA content; (**d**) number of seeds per silique; (**c**) 100-seed weight; (**e**) total FA content; (**f–h**) quantitative real-time PCR analysis of *AtDGAT1* (**f**); *AtGPAT9* (**g**); and *AtOleosin* (**h**) expression levels in *PrLPAAT4*-overexpressing transgenic Arabidopsis plants. *Error bars* represent the mean ± SD of three independent biological replicates. *Atactin7* was used as a reference gene. *Asterisks* denote significant difference using Student’s *t* test. * indicates *p* < 0.05; ** indicates *p* < 0.01.

To better dissect the function of *PrLPAAT4*, transgenic Arabidopsis plants expressing *PrLPAAT4* were generated. Three independent homozygous *T*_2_ lines, L4OX-12, L4OX-22 and L4OX-25, exhibiting the greatest expression of *PrLPAAT4*, were confirmed via semi-quantitative RT-PCR (Figure 6a). In addition, compared with wild-type (WT) plants, the average of 100-seed weight and number of seeds per silique had a significant increase changed in *PrLPAAT4*-overexpressing lines (Figure 6c,d).

Furthermore, to study the role of *PrLPAAT4* in FA biosynthesis, we analyzed the content and composition of FAs in mature seeds of the transgenic Arabidopsis plants overexpressing *PrLPAAT4* using GC-MS. We found that the total FAs content of the seeds in T_2_ lines L4OX-12, L4OX-22 and L4OX-25 were 5.8%, 7.1% and 7.8%, respectively higher than that in WT control (Figure 6e and Table S3). To further determine that the increase in the total FAs content was caused by which specific FA, we assessed the abundance of the main FA components in WT and transgenic seeds. By comparison with WT, the levels of C18:1 (oleic acid) were significantly increased in the transgenic seeds of three lines (Figure 6b and Table S3).

To clarify the molecular mechanism of *PrLPAAT4* in FA biosynthesis and TAG assembly, we detected the expression levels of *DGAT* and *GPAT*, as well as the gene encoding the major oil body protein (*Oleosin*). Compared with control line, transcript levels of *AtDGAT1*, *AtGPAT9*, and *AtOleosin* were significantly increased in transgenic Arabidopsis lines overexpressing *PrLPAAT4* (Figure 6f–h).

## 3. Discussion

Currently, LPAATs have been extensively explored for functional and biotechnological studies. Microsomal LPAATs are classified into at least two classes, class A and B, based on the difference in subcellular localization. In this study, we identified a *PrLPAAT4* gene encoding a LPAAT-like protein from the tree peony. Subcellular localization of PrLPAAT4 in the lower epidermis cells of tobacco leaves indicated that this protein targeted the plasma membrane (Figure 5), which was consistent with our predicted result by PSORT Prediction and subcellular localization of AhLPAAT4 from *A. hypogaea* [15].

Phylogenetic analysis further demonstrated the evolutionary relationship between PrLPAAT4 and LPAAT4 proteins from other plant species. PrLPAAT4 shared high similarity with the homologues proteins from *C. clementina*, *P. trichocarpa*, *M. esculenta*, and *R. communis* (Figure 3), indicating that these proteins probably play similar roles in plant lipid biosynthesis. Additionally, phylogenetic analysis showed three main distinct deep branching clusters of LPAAT proteins. The clusters were designated as cluster I, cluster II, and cluster III, respectively, with cluster I and III being dicots, and cluster II being monocots. There is a high structural conservation among proteins from each cluster. These results indicate that LPAAT4s exist early in plants and are transmitted both vertically and horizontally during the eukaryotic evolution.

Polypeptidesequence analysis of PrLPAAT4 revealed four highly conserved motifs (I-IV), which are important to acyltransferase reactions (Figure 1). In the AGPAT family, all enzymes possess the HXXXXD signature and the majority harbors the PEGT-X signature [31]. Specifically, acyltransferase motifs I and III are the most conserved regions with invariant residues [31,32], and they are involved in the catalyzation of acyltransferase activity [33] and the binding of the acyl acceptor [31], respectively. Therefore, we hypothesized that PrLPAAT4 had acyltransferase activity and possibly contributed to glycerolipid metabolism in plant cells. Moreover, PrLPAAT4 also contained the specific residues in motif II and IV, which may be responsible for the binding of acyl acceptor LPA and G3P [31], and acyl-CoA binding [34]. Thus, we proposed that PrLPAAT4 may be a member of the AGPAT family.

In the present study, three independent homozygous lines showed markedly different transcript abundances of *PrLPAAT4* (Figure 6a). It is probably caused by the integration of *PrLPAAT4* fragment into different loci of the chromosomal DNA in three transgenic Arabidopsis lines. Previous studies have indicated that quantitative variation in exogenous genes expression among individual transformants is generally ascribed to different integration sites of exogenous genes [35,36,37,38]. Transgenic Arabidopsis plants showed that the average of 100-seed weight and number of seeds per silique were significantly increased (Figure 6c,d). Apart from seed yield-related traits, the seed quality-related ones were also examined in the transgenic plants. In plants, the content of specific fatty acid can be accumulated due to the selectivity of LPAAT [39,40]. *PrLPAAT4* and *PrLPAAT1* individually may be more specific to C18:1 and C18:2 because the findings that overexpression of *PrLPAAT4* and *PrLPAAT1* led to a significant increase in C18:1 (Figure 6b) and C18:2, respectively [29]. Many reports have shown that the fatty acid pattern of TAG usually is primarily controlled by the acyl-CoA specificities and selectivities of the microsomal LPAATs [41]. During TAG assembly, the LPAAT has the highest substrate stringency and dictates [42]. Additionally, LPAAT possesses strong preference for unsaturated fatty acids [43,44], especially for C18 acyl groups [4]. The *Brassica* LPAAT shows a preference for C18:1 [45]; the preferred substrate of flax LPAAT is C18:2-CoA. In addition, the meadowfoam LPAAT is highly active towards LPA-22:1 and erucoyl CoA [40]; the LPAAT of *Cocos nucifera* displays high activity for medium-chain-length substrate specificity [11]. We speculate that the members of the LPAAT family of the same species and the LPAATs of different species may both have specificity and selectivity for FAs. In addition, the result that *PrLPAAT4* overexpression in Arabidopsis resulted in the improvement in the total FAs content (Figure 6e) is consistent with the findings from many previous reports. Overexpression of peanut *AhLPAAT2* in Arabidopsis, the total FA content of the seeds significant increased [46]. The total FAs content is increased in the *PrLPAAT1*-overexpressing Arabidopsis plants [29]. Similar results can be found in Arabidopsis [19] and rapeseed [17]. All results indicate that different plants *LPAAT*s have complex effects on the synthesis and content of FA, and require further study and discussion. It is highly likely that *PrLPAAT4* overexpression could result in an improvement in seed yield and quality traits, providing a theoretical foundation for the seed oil-related molecular studies of tree peony and other oil crops.

We found that the genes, *AtGPAT9*, *AtDGAT1*, and *AtOleosin* involved in TAG assembly via Kennedy pathway displayed higher expression levels in *PrLPAAT4*-overexpressing Arabidopsis plants than WT (Figure 6f–h). The same result was found in transgenic Arabidopsis overexpressing *AhLPAT2* from *A*. *hypogaea* [46]. Nevertheless, the molecular mechanism of the result remains unknown and need further studies. It seems likely that the expression of transgenes could affect the normal expression patterns of corresponding endogenous genes as well as genes involved in the same or related processes. Overall, there may be a positive feedback mechanism that an increase in PrLPAAT4 activity causes the upregulation of the genes related to TAG assembly. However, the molecular mechanism of PrLPAAT4 for FA biosynthesis in tree peony still remains unclear, and requires further examination in future work.

## 4. Materials and Methods

### 4.1. Plant Materials

The seeds of *P. rockii* were harvested in Wild Tree Peony Repository at Northwest A&F University (Yangling, China) between April and July. The seeds at 20, 30, 40, 50, 60, 70, 80, 90 and 100 days after flowering (DAF) and other tissues, including roots, topmost stems, younger leaves, calyxes, petals, stamens, and pistils of *P. rockii* flowers at anthesis, were hand-collected for gene expression analysis. All samples with three biological replicates were taken from at least three plants, and were frozen in liquid nitrogen immediately and stored at −80 °C until further use.

### 4.2. Genomic DNA and Total RNA Isolation

Genomic DNA was extracted from the *P. rockii* seeds at 30 DAF using the hexadecyltrimethylammonium bromide (CTAB) method with some minor modifications [47]. Total RNA was isolated using the TIANGEN RNA Prep Pure Plant kit according to the manufacturer’s instructions (Tiangen Biotech Co. Ltd., Beijing, China). The quality and concentration of RNA samples were tested by Goldview-stained agarose gel electrophoresis and spectrophotometric analysis, respectively. Total RNA samples were reverse-transcribed to generate the first-strand cDNA using PrimeScript^®^ RT reagent Kit with gDNA Eraser (DRR047A, Takara, Dalian, China).

### 4.3. Full-Length Cloning of PrLPAAT4

The conserved fragments of *PrLPAAT4* were PCR-amplified from cDNA templates using degenerate primers composed of two sense primers and two antisense primers (Table S1). The sequence amplification of 5′ and 3′ ends of *PrLPAAT4* cDNA was performed based on Rapid Amplification of cDNA Ends (RACE) technique using SMARTer^®^ RACE 5′/3′ Kit (Clontech Laboratories, Inc., Mountain View, CA, USA), following the manufacturer’s instructions. To obtain the coding region of *PrLPAAT4*, specific primers (Table S1) spanning full open reading frames (ORFs) were designed. All PCR amplification products were cloned into pUCm-T vetor (SK2212, Sangon, Shanghai, China) for sequencing.

### 4.4. Sequence and Phylogeny Analysis

Homology search of sequences at nucleotide and protein levels was carried out through Blast (http://blast.ncbi.nlm.nih.gov). Phylogenetic tree was constructed using MEGA (version 5.1) software [48], based on multiple sequence alignment by MUSCLE (http://www.ebi.ac.uk/Tools/msa/muscle/). The hydrophobic property and charge distribution were analyzed using ExPASy (http://www.expasy.org/). The protein molecular weight and isoelectric point were evaluated using ProtParam software (http://web.expasy.org/protparam/). The secondary and tertiary structure predictions were conducted using SOPMA (https://npsa-prabi.ibcp.fr/cgi-bin/npsa_automat.pl?page=/ NPSA/npsa_sopma.html) and Phyre Version 0.2 (Imperial College London, South Kensington Campus, London SW72AZ, UK) [49], respectively. The putative subcellular localization was performed using PSORT Prediction (http://psort.hgc.jp/form.html). The sizes of exon and intron of *PrLPAAT4* DNA were measured, and the structural models of gene and protein were drawn with FancyGENA v1.4 software (Eurpean Institute of Oncology, IFOM-IEO Campus, Via Ademello 16, 20139 Milan, Italy) [50]. The transmembrane prediction server TMHMM2.0 (http://www.cbs.dtu.dk/services/) and the SMART database (http://smart.emblheidelberg.de/) were used to identify the conserved transmembrane domains (TMDs) of PrLPAAT4.

### 4.5. Subcellular Localization

The *PrLPAAT4* ORF region without the stop codon was inserted into the *BamH* I-*Sal* I restriction sites of binary vector pC1301-*GFP* to generate 35S::*PrLPAAT4*-*GFP* construct. The constructed plasmid was transformed into the *Agrobacterium tumefaciens* strain EHA105 by electroporation. Positive colony was selected on an LB plate supplemented with 50 mg/mL rifampicin and kanamycin, and cultured in liquid LB medium containing appropriate antibiotics at 28 °C in dark until OD600 of 0.3 for the final cell density. After centrifugation and cell collection, the pellets were resuspended in the same volume of infiltration buffer (10 mM MES, 200 μM acetosyringgone, and 10 mM MgCl_2_, pH 5.6), by shaking at room temperature for 4–6 h. Afterwards, the construct was delivered into the lower epidermis of 4-week-old tobacco leaves by agro-infiltration. The GFP fluorescence was observed at 4–6 days post-transfection using the UltraVIEW Vox spinning disk confocal system (PerkinElmer, Cambridge, UK).

### 4.6. Overexpression Construct and Stable Transformation

The coding sequence of *PrLPAAT4* was ligated into the *Kpn* I-*Xba* I restriction sites of binary vector pCAMBIA1300 under the control of Arabidopsis seed-specific promoter 2S2. The generated 2S2::*PrLPAAT4* construct was transformed into wild-type Arabidposis plant (WT) using the floral dip method as previously described [51]. The harvested seeds were planted on 1/2 MS plates containing 20 mg/L hygromycin in Sanyo MLR 351H growth chamber at 22 ± 2 °C under a 14/10 h light/dark (120 µmol m^−2^·s^−1^) cycle. Hygromycin-resistant seedling with green leaves and well-established roots were selected as *T*_0_ transformants, and followingly transferred from the plates to moistened potting soil. The positive transformants was confirmed via the PCR method. Independent transgenic Arabidopsis lines exhibiting a 3:1 segregation of hygromycin (20 mg/L) resistance in the *T*_1_ generation were selected. Homozygous *T*_2_ transgenic lines showing 100% survival on hygromycin-containing medium were finally established. Mature Arabidopsis seeds were collected from *T*_2_ homozygous lines for further analysis.

### 4.7. FA Measurement

Twenty milligrams of dried Arabidopsis seeds were collected from ten plants, comprising three replicates. We followed the extraction protocol for FAs and the preparation of FA methyl esters as described previously [52]. FA analyzed by using a gas chromatograph-mass spectrometer (GC68990N/MS5937, Aglient Technologies, Santa Clara, CA, USA) equipped with a G4513A autosampler (Agilent). The column was an aryl-polysiloxane packed capillary column (HP-88; 30 m × 0.25 mm i.d., 0.20 µm film thickness; Agilent). Qualitative FA analysis was achieved by a mass spectra database search (NIST08 Library) and co-elution with corresponding standards. A standard curve method with an internal standard was used as the quantitative approach to construct five calibration plots of analyte/internal standard peak-area ratio versus standard concentration, as determined by the least squares method. The FAMEs in each sample were measured using methyl heptadecanoate as the internal standard. The FAMEs were expressed as milligrams per gram dry weight (DW) of sample. All samples were analyzed in triplicate under the same conditions.

### 4.8. Quantitative Real-Time PCR

The seeds of WT and the homozygous *T*_2_ line of transgenic Arabidopsis at 14 DAF were used for expression analysis. The isolation of total RNA and synthesis of the first-strand cDNA were performed as described above. *Atactin7* was used as reference gene to normalize the expression data. Quantitative real-time PCR (qRT-PCR) was conducted using Premix Ex Taq^TM^ (Perfect Real Time) kit (DRR041A, Takara, Dalian, China) in a LightCycler480 Real-Time PCR System (Roche Diagnostic, Basel, Switzerland). All samples were analyzed with three biological replicates. Relative expression levels were computed using the 2^−△△*C*T^ comparative threshold cycle (*C*t) method [53]. All primers used for the assessment of transcript levels were listed in Table S1.

### 4.9. Statistical Analysis

All experiments included three biological replicates and technical replicates as previously indicated. Mean ± SD were determined and one-way analysis of variance was carried out using SPSS (version 17.0 for Windows; Chicago, IL, USA), to determine significance at *p*-value < 0.05.

## Supplementary Materials

Supplementary Materials are available online. Table S1 Primers used in this study, Table S2 Proteins used for construction of phylogenetic tree, Table S3 Fatty acid contents in mature wild-type and *PrLPAAT4*-overexpressing transgenic Arabidopsis seeds.
