# Supplementary material for: PrLPAAT4, a Putative Lysophosphatidic Acid Acyltransferase from Paeonia rockii, Plays an Important Role in Seed Fatty Acid Biosynthesis
Source: Molecules. 2017 Oct 10;22(10):1694. doi: 10.3390/molecules22101694 (PMC6151692; doi:10.3390/molecules22101694)
Supplement: Supplementary file 1 [file molecules-22-01694-s001.pdf]

Table S1. Primers used in this study.

Name	Primer sequences (5'- 3')	Application
dp-S1	GRACNGARGTKGAYTGG	Degenerated primers for amplification of the conserved region
dp-S2	ACNGARGTKGAYTGGATG	
dp-A1	TCRTAWACTGCRTCMADDGA	Degenerated primers for amplification of the conserved region
dp-A2	AAATCDGTBCCYTCVGGG	
LPAAT4-1F3（GSP1）	GATTACGCCAAGCTTACTGAGGTKGATTGGATG	3'RACE
LPAAT4-2F3（NGSP1）	GATTACGCCAAGCTTATTCCCTCTGGCTTGCTC	
LPAAT4-1R5（GSP1）	GATTACGCCAAGCTTGGAAAAGAGCAAGCCAGAGGG	5'RACE
LPAAT4-2R5（NGSP1）	GATTACGCCAAGCTTCAACTTCCCACCTCCTCTCC	
LPAAT4F	GATCAGATGGAAGTTCGAGGGC	Full-length cDNA PCR
LPAAT4R	CAGAATAGTTTACRCCGCATACC	
LPAAT4F	GATCAGATGGAAGTTCGAGGGC	Full-length DNA PCR
LPAAT4R	CAGAATAGTTTACRCCGCATACC	
LPAAT4RT-F	CAATGTCTACGGAGTGGAACC	Semi-quantitative RT-PCR
LPAAT4RT-R	CGACGAAAAAAATGTGAGGAAC	
AtGPAT9-F	TCGGAAACCGGCGACGTAAGC	qRT-PCR
AtGPAT9-R	TGGCACCAGCAGCTTCAGTGAG	
AtDGAT1-F	TGGAGCTCCCGCCGACGTTA	qRT-PCR
AtDGAT1-R	TCGCCCTCCGATGAGCTGGA	
AtOlesin-F	TCCTTTATCCTCAGAGTGGCCCTT	qRT-PCR
AtOlesin-R	AAGGCTGCTGGTACAATAACCGGA	
Atactin7-F	GGAACTGGAATGGTGAAGGCTG	Semi-quantitative RT-PCR and qRT-PCR
Atactin7-R	CGATTGGATACTTCAGAGTGAGGA	

Table S2. Proteins used for construction of phylogenetic tree.

Family	Specie	Taxa	Protein ID	Length (aa)	No. Introns	Database
Euphorbiaceae	*Manihot esculenta*	Mes	cassava4.1_009731m	379	2	Phytozome
			cassava4.1_024286m	345	2	Phytozome
Euphorbiaceae	*Ricinus communis*	Rco	29851.m002448	356	3	Phytozome
Salicaceae	*Populus trichocarpa*	Ptr	Potri.002G133100	372	2	Phytozome
			Potri.014G040600	386	2	Phytozome
			Potri.009G112300	368	2	Phytozome
Fabaceae	*Glycine max*	Gma	Glyma12g04980	375	2	Phytozome
			Glyma11g12830	375	2	Phytozome
			Glyma17g36670	383	2	Phytozome
			Glyma14g08400	383	2	Phytozome
Brassicacea	*Arabidopsis thaliana*	Ath	AT1G75020	378	2	Phytozome
Brassicacea	*Arabidopsis lyrata*	Aly	476640	379	2	Phytozome
Rutaceae	*Citrus sinensis*	Csi	orange1.1g016832m	382	2	Phytozome
Rutaceae	*Citrus clementina*	Ccl	Ciclev10001493m	382	2	Phytozome
			Ciclev10028764m	343	2	Phytozome
Myrtaceae	*Eucalyptus grandis*	Egr	Eucgr.G00474	379	2	Phytozome
Vitaceae	*Vitis vinifera*	Vvi	GSVIVT01009645001	379	2	Phytozome
Ranunculaceae	*Aquilegia coerulea Goldsmith*	Aco	Aquca_007_00141	383	2	Phytozome
			Aquca_010_00407	397	2	Phytozome
Poaceae	*Sorghum bicolor*	Sbi	Sb09g024780	406	2	Phytozome
			Sb03g036470	399	2	Phytozome
Poaceae	*Zea mays*	Zma	GRMZM2G014981	403	2	Phytozome
			GRMZM2G135027	399	2	Phytozome
Poaceae	*Setaria italica*	Sit	Si022242m	399	2	Phytozome

			Si003996m	398	2	Phytozome
Poaceae	*Oryza sativa*	Osa	LOC_Os01g57360	399	2	Phytozome
			LOC_Os05g42270	397	2	Phytozome
Poaceae	*Brachypodium distachyon*	Bdi	Bradi2g20650	399	2	Phytozome

Table S3. Fatty acid contents in mature wild-type and *PrLPAAT4*-overexpressing transgenic Arabidopsis seeds (mg g^{-1}DW, mean ± SD, n = 3).

	WT	L4OX-12	L4OX-22	L4OX-25
C16:0	19.98691 ±0.752651	20.8841±0.294714	20.41389 ± 1.114745	21.29916±0.912885
C16:1	3.716335± 0.388068	3.229979 ±0.638296	3.763577 ± 0.389939	4.427175 ± 1.005644
C18:0	7.550231 ±0.338142	7.585593 ± 0.368444	7.862527± 0.184257	7.655451± 0.101081
C18:1	66.42992 ± 2.13838	80.42564 ± 6.247013	77.54157 ± 1.41665	81.72358±4.879747
C18:2	85.95135 ± 1.837197	88.82653 ± 5.98847	86.47376 ± 2.285479	91.52029±2.278007
C18:3	59.42794± 1.954724	57.28866 ± 2.890718	64.19366 ± 1.570903	58.48088± 2.709316
C20:0	4.305935 ± 0.035865	3.762568 ± 0.379518	3.96285 ± 0.303509	3.963452±0.187219
C20:1	39.28514 ± 2.645662	41.78146 ±1.383087	43.41023 ± 0.845402	41.07181± 1.908515
C20:2	3.720144 ± 0.331264	3.31346 ± 0.312434	3.597367± 0.367573	3.37524± 0.202573
C20:3	0.385623264± 0.123678587	0.611946689± 0.401983771	0.686161536 ±0.111196637	0.594675375 ±0.099064611
C22:1	3.590458 ±0.082721	3.606877 ± 0.362823	3.414285 ± 0.33179	3.286447±0.182905
Total FAs	294.35 ± 6.77094	311.3169 ± 5.523421	315.3199 ± 4.76069	317.3982±4.501371
